# Interfacial cavitation

**DOI:** 10.1093/pnasnexus/pgac217

**Published:** 2022-10-03

**Authors:** Thomas Henzel, Japinder Nijjer, S Chockalingam, Hares Wahdat, Alfred J Crosby, Jing Yan, Tal Cohen

**Affiliations:** Department of Civil and Environmental Engineering, Massachusetts Institute of Technology, Cambridge, MA 02139, USA; Molecular, Cellular and Developmental Biology, Yale University, New Haven, CT 06520, USA; Department of Aeronautics and Astronautics, Massachusetts Institute of Technology, Cambridge, MA 02139, USA; Polymer Science and Engineering Department, University of Massachusetts Amherst, Amherst, MA 01003, USA; Polymer Science and Engineering Department, University of Massachusetts Amherst, Amherst, MA 01003, USA; Molecular, Cellular and Developmental Biology, Yale University, New Haven, CT 06520, USA; Quantitative Biology Institute, Yale University, New Haven, CT 06520, USA; Department of Civil and Environmental Engineering, Massachusetts Institute of Technology, Cambridge, MA 02139, USA; Department of Mechanical Engineering, Massachusetts Institute of Technology, Cambridge, MA 02139, USA

## Abstract

Cavitation has long been recognized as a crucial predictor, or precursor, to the ultimate failure of various materials, ranging from ductile metals to soft and biological materials. Traditionally, cavitation in solids is defined as an unstable expansion of a void or a defect within a material. The critical applied load needed to trigger this instability -- the critical pressure -- is a lengthscale independent material property and has been predicted by numerous theoretical studies for a breadth of constitutive models. While these studies usually assume that cavitation initiates from defects in the bulk of an otherwise homogeneous medium, an alternative and potentially more ubiquitous scenario can occur if the defects are found at interfaces between two distinct media within the body. Such interfaces are becoming increasingly common in modern materials with the use of multimaterial composites and layer-by-layer additive manufacturing methods. However, a criterion to determine the threshold for interfacial failure, in analogy to the bulk cavitation limit, has yet to be reported. In this work, we fill this gap. Our theoretical model captures a lengthscale independent limit for interfacial cavitation, and is shown to agree with our observations at two distinct lengthscales, via two different experimental systems. To further understand the competition between the two cavitation modes (bulk versus interface), we expand our investigation beyond the elastic response to understand the ensuing unstable propagation of delamination at the interface. A phase diagram summarizes these results, showing regimes in which interfacial failure becomes the dominant mechanism.

Significance Statement A fundamental question when working with any solid material is: when will it fail? In this work, we report a hitherto unconsidered failure mechanism “interfacial cavitation” and provide experimental proof to confirm that it is prevalent in both natural and synthetic material systems. Though cavitation that initiates from defects in the bulk has been documented in solids since the 50s and is by now established as a primary mode of failure initiation in a wide range of solids, from hard metals to soft and biological materials, a criterion to determine an analogous scale-free threshold for interfacial cavitation has yet to be reported. In this work, we fill this gap.

The genesis of the study of cavitation dates back to the seminal work of Rayleigh in 1917 (1). (Rayleigh mentions the earlier work by Besant (2), which considered the same problem but did not resolve the internal pressure.) Concerned with the growth and subsequent collapse of bubbles in water, Rayleigh proposed a simple model that estimates the internal pressure in a collapsing spherical cavity. Since then, the violent collapse of bubbles that form near solid surfaces has been studied extensively.

Perhaps the earliest study of cavitation in solids dates back to the work of Bishop, Hill, and Mott in 1945 (3), which aimed to obtain theoretical predictions for interpretation of indentation and hardness tests in ductile metals. They argued that the maximum resisting pressure attained in the indentation process can be well estimated by the pressure required to expand a cavity indefinitely. Though it is not obvious that a constant finite pressure can induce indefinite expansion, their theory predicted that such an asymptotic pressure exists and is lengthscale independent; it is thus a material property—the *cavitation pressure*. The notion that this material property is useful to determine the ability of a solid to sustain loads has since been extended beyond indentation. By now, it is well established as a criteria for onset of ductile fracture (4–9), it has recently been indicated as an underlying mechanism of failure in brittle materials (10, 11), and it serves as a measure for estimation of static and dynamic penetration and perforation processes (12–16). Additionally, in recent years, the cavitation pressure has been pivotal in modern methods to measure properties of soft and biological materials (17–21), and its use for predicting the failure of rubbers has been subjected to an ongoing debate (22–25). Moreover, the long-established neo-Hookean cavitation pressure, *p_bc_*/μ = 5/2, for incompressible materials with shear modulus μ, has become foundational in explaining chemical and biological processes in which cavities can form spontaneously inside the material (26–29).

Despite the vast body of literature on cavitation in solids that has accumulated over the years (22,30–36) and in contrast to the study of cavitation in fluids, theoretical predictions in solids commonly consider cavitation that initiates from defects in the bulk of an otherwise homogeneous material, while cavitation that initiates from interfaces is only rarely mentioned (37–41). Nonetheless, defects are often found around impurities and at interfaces, and thus generating cavities far from interfaces can be challenging (24). Moreover, with the growing application of composites that integrate multiple materials as well as layer-by-layer additive manufacturing methods, the use of materials that are prone to failure at interfaces is becoming increasingly ubiquitous; in these materials, initiation of failure from cavitation at interfaces may dominate over cavitation in the bulk. There are several possible explanations for the existing gap in providing a theory for interfacial cavitation that is as simple and elegant as cavitation in the bulk (30,31, 33, 34). When a defect is embedded in the bulk of a material, its expansion can be well described using simplifying assumptions on the symmetry of the deformation field. This luxury must be forfeited when attempting to consider cavities at an interface, thus requiring a new approach to define the problem setting, and the aid of computational tools to capture the expansion process deep into the nonlinear range of the material response. Most importantly, it is not obvious that an asymptotic pressure even exists and if the lengthscale independent property of bulk cavitation translates to interfacial cavitation. Finally, a major driver of earlier studies on cavitation has been their observation (30, 42, 43). However, interfacial failure, is rarely considered from the viewpoint of cavitation, and has instead been interpreted as an interfacial fracture and delamination process (44–49). Some studies use the bulk cavitation limit as an approximation for interfacial cavitation (50, 51), however report that it underestimates the critical value (52) and acknowledge the need for models that can capture the nonlinear deformation near a substrate.

To remedy this gap, our work presents (i) a complete theoretical framework that is analagous to bulk cavitation, thus exposing a lengthscale independent interfacial cavitation limit and capturing the ensuing delamination process; (ii) a phase diagram that determines the competition between bulk and interfacial cavitation and the stability thresholds across a broad range of normalized pressures and interface properties; and (iii) observations of interfacial cavitation and delamination for a direct comparison and validation of the theory.

To establish interfacial cavitation as a lengthscale independent process, we exploit two experimental systems. At the small scale, with defects of the order of ∼10 μm, we examine the interstitial growth of biofilm, starting from one *Vibrio cholerae* bacterium embedded at the interface between a soft material (agarose) and a glass substrate to which it is bonded. In this highly controlled growth process, the evolving biofilm behaves like an expanding fluid as it proliferates (53), allowing the tracking of morphological changes during growth (54). At larger scales of ∼100 μm, we exploit the Pressurized Interfacial Failure (PIF) experimental set-up, recently described in (39), whereby interfacial separation is controlled by applying fluid pressure at a localized region of a bonded interface.

## An asymptotic interfacial cavitation pressure

A key characteristic of the bulk cavitation pressure is that it is universal in the sense that it does not depend on the size of the defect. This lengthscale independence arises from the assumption that the defect is small compared to the size of the body, and neglects the effect of surface tension. [It has been shown that if surface tension is present, it can resist cavitation, thus making larger defects more prone to the cavitation instability (30,52).] Using the same basic assumptions, in this work, we consider a semi-infinite body that is bonded to a rigid substrate. We prescribe a defect as being a circular region of the interface, of diameter *l*, that is not bonded. To establish the existence of a critical interfacial cavitation pressure, we first assume that other regions of the interface are strictly bonded; the consequence of relaxing this assumption will be considered in the next section. Accordingly, we define our cartesian coordinate system (*X, Y, Z*) with its origin at the center of the defect, such that the undeformed body occupies the region (*X, Y*) ∈ ( −∞, ∞), *Z* ∈ [0, ∞), and with the defect in the range $X^2 + Y^2 \leq l^2/4$, *Z* = 0. We assume that axial symmetry is preserved as the interfacial cavity expands, which is also supported by our experimental observations.

The analysis presented in this paper can be conducted using any constitutive model of choice. Nonetheless, to compare our results with the well-established neo-Hookean bulk cavitation pressure, we limit our attention to incompressible neo-Hookean response. Thus, we have the free energy function ψ = μ(*I*_1_ − 3)/2, where *I*_1_ is the first invariant of the left Cauchy-Green deformation tensor.

As in the case for bulk cavitation, there are two ways to expand the cavity. By application of internal pressure at the cavity wall, or by application of remote tension. For bulk cavitation in incompressible materials, analytical derivations show that the cavitation pressure is the same for both scenarios (55, 56). The nonlinear reciprocal theorem (57) confirms that the same is true also for interfacial cavitation. Hence, in our finite element simulations, we quasistatically expand the interfacial cavity by application of pressure at the wall of the defect. This procedure is implemented in the Finite Element (FE) software ABAQUS/CAE 2017. To eliminate boundary effects, we set the size of the domain to 100 times the initial length of the defect and we have confirmed that changes in the remote field remain negligible. Quadratic, axisymmetric, and hybrid elements (CAX8H) are used to capture large axisymmetric deformations and incompresibility. The mesh is highly refined around the defect, such that the length of the smallest element is *l*/10^5^.

**Fig. 1.**
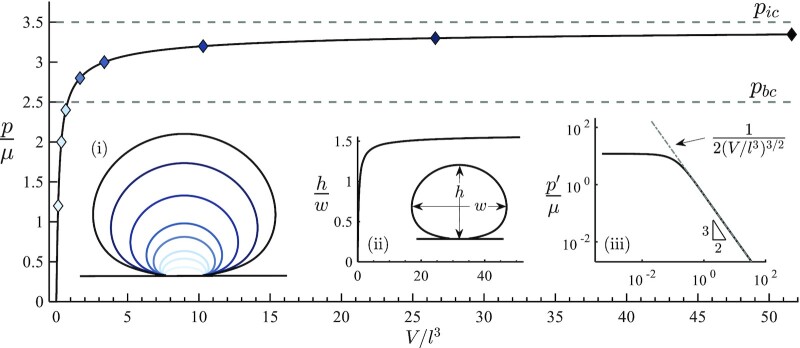
The applied cavity pressure approaches an asymptotic limit of *p_ic_*/μ → 7/2 with increasing volumes. The shape of the cavity cross-section at different dimensionless expansion volumes is shown in inset (i), where curve shades correspond to the diamond markers along the pressure–volume curve. The aspect ratio of these shapes approaches an asymptotic value of *h*/*w* ∼ 1.6, as shown in inset (ii). The power law decay of the slope of the pressure–volume curve, shown via the log–log plot in inset (iii), confirms the asymptotic behavior. The data set for the pressure–volume curve and a video of the simulated expansion process can be found in the Supplementary Material.

As shown in Fig. 1, our simulation reveals that the normalized interfacial cavity pressure (*p*/μ) approaches an asymptotic value of *p_ic_*/μ ∼ 7/2 that is analogous to that obtained for cavity expansion in the bulk. Our analysis confirms that this trend is indeed asymptotically approaching a finite value. In addition to refining our computations to access pressures in the range of extreme deformations with a volume expansion *V* as large as 50*l*^3^, we consider the evolution of its slope $p^{\prime} = dp/d(V/l^3)$ on a log–log plot and find that it decays like (*V*/*l*^3^)^−3/2^. Assuming this asymptotic decay, for large dimensionless volumes *V*/*l*^3^ ≫ 1, the evolution of pressure takes the simple form $p\cong p_{ic}-\mu /\sqrt{V/l^3}$.

In examining the evolving shape of the cavity as it expands, we find that, in contrast to bulk cavitation (58), a self-similar field does not appear to emerge, even deep into the nonlinear range. Nonetheless, the aspect ratio of the cavity saturates at a value of ∼1.6, and does not tend towards a spherical shape. This limiting value of the aspect ratio can be used to determine whether an experimental observation is approaching the cavitation limit and to thus infer whether the pressure within the cavity is approaching *p_ic_*. A video of the expansion process is provided in the Supplementary Material, where the stress concentration is shown by the darker shades, which represent variation in the second invariant of the deviatoric stress tensor field.

We anticipate that by considering a rigid substrate, the interfacial cavitation pressure obtained here serves as an upper bound for the case of deformable substrates, whereas the case of a substrate that has stiffness identical to that of the body will cavitate at a pressure that is well represented by the spherical cavitation limit. This is confirmed by earlier studies that show that the initial shape of the defect has little influence on the cavitation pressure (20). Nonetheless, additional work is needed to elucidate the relationship between the stiffness ratio of the two materials, and the resulting interfacial cavitation pressure, which is beyond the scope of this work.

It should be noted that the extreme deformations that are achieved in the vicinity of the cavity, as the asymptotic value of the cavitation pressure is approached, are unlikely to be well represented by a neo-Hookean model. This is true also in the case of bulk cavitation. Nonetheless, the internal pressure is determined by the resistance of the entire field, and thus the neo-Hookean model is considered a good approximation, even if some inelastic effects occur in a localized region near the cavity wall. A competition between different instability modes may emerge, be it cavitation in the bulk, at the interface, or alternatively failure of the interfacial bond, as discussed next. [In this work, we do not consider the possible onset of fracture in the bulk of the material, which has been the focus of an earlier study (25).]

## Unstable cavity growth

The presence of an asymptotic value of the interfacial pressure, as shown in Fig. 1, implies that upon approaching *p_ic_*, small perturbations of pressure can induce substantial changes in cavity volume, namely unstable growth. By comparing interfacial cavitation with bulk cavitation, we find that locally pressurized interfacial cavitities can withstand pressures that would “rip the material apart” if applied in the bulk. This striking $40 \%$ increase in normalized critical pressure (i.e. *p_ic_*≅1.4*p_bc_*) can be attributed to the additional strength that is provided by the rigid constraint of the substrate. However, if the strength of the interfacial bond is finite, failure of the interface may initiate first.

To examine the possible onset of interfacial failure, we consider the energetically favorable states of the system for a given prescribed volume. The system is characterised by the two independent state variables: the dimensionless volume (*V*/*l*^3^) and the defect length (*l*). Central to our formulation is the fact that the elastic expansion process is length scale insensitive for the assumption of semi-infinite body and that the relationship between the dimensionless pressure and the dimensionless volume, plotted in Fig. 1, applies for cavities of any defect length. Accordingly, we write the relationship between the dimensionless pressure and the dimensionless volume in Fig. 1 as $p/\mu=f^{\prime}(V/l^3)$, where the prime denotes differentiation. The total elastic energy in the system can thus be directly written as $E_e=\int _0^V p{\rm d} V=\mu l^3 f(V/l^3)$.

If delamination is permitted, an additional energetic cost would be incurred to create new surface area, which we write as $E_d=\Gamma \frac{\pi }{4}(l^2-l_0^2)$, where *l*_0_ denotes the initial defect diameter, and Γ > 0 is the energy needed to debond a unit area of the surface—the interface toughness. The total energy invested in expanding the cavity is thus the sum of the two contributions *E_t_* = *E_e_* + *E_d_*, which can be written in its dimensionless form $\mathcal {E}_t=E_t/(\Gamma l_0^2)$ as

(1) $$\begin{equation*}
\mathcal {E}_t(l;l_0,V)= \varphi \left(\frac{l^3}{l_0^3}\right) f(V/l^3)+ \frac{\pi }{4} \left(\frac{l^2}{l_0^2}-1\right),
\end{equation*}
$$

where the dimensionless model parameter φ = μ*l*_0_/Γ determines the balance between elastic and interfacial effects. For a prescribed volume *V*, the energy in Eq. (1) depends only on the deliminated size of the defect *l*, which in an equilibrium configuration will minimize the total energy. [Note that this energetic argument only holds for the forward process of delamination. Upon unloading, additional considerations may apply in describing the re-engagement of the two surfaces (38).] Formally, this can be written as

(2) $$\begin{equation*}
l={\rm arg}~ \underset{l}{\rm min} \,\,\left(\mathcal {E}_t(l;l_0,V,\varphi ) \right),
\end{equation*}
$$

which can be evaluated by taking the derivative, such that ${\partial }\mathcal {E}_t/{\partial }l=\varepsilon _t(l;l_0,V,\varphi )=0$, and the corresponding pressure is obtained from the pressure volume curve (Fig. 1). By analyzing this functional form, we find that the onset of interfacial failure is a second order transition. We can thus evaluate the critical conditions for initiation of failure by substituting *l* = *l*_0_, to write ε_*t*_(*l*_0_; *l*_0_, *V*, φ) = 0, which provides us with an implicit relationship between the normalized critical volume and the model parameter, $V_c/l^3_0=g(\varphi )$. The corresponding critical pressure is then obtained as $p_c(\varphi)/\mu = f^{\prime}(g(\varphi))$.

**Fig. 2.**
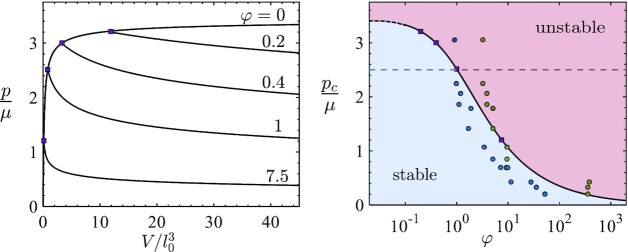
The dimensionless material property φ = μ*l*_0_/Γ determines the stability threshold. On the left, departure of the pressure a from the purely elastic response is shown for various values of φ, and indicted by the square markers. The corresponding critical pressure (*p_c_*/μ) is shown as a function of φ on the right, to form a phase diagram, with the square markers corresponding to the curves on the left. In the blue region the response is stable; in the red region perturbations can lead to unstable expansion. The dashed line corresponds to the bulk cavitation pressure. The circular markers represent critical pressures measured in different materials using the PIF method, where the interface toughness is determined via conventional probe-tack test (blue) or linear theory (39) (green). Experimental details can be found in the Supplementary Material.

Fig. 2 shows the evolution of pressure with increasing cavity volume for various values of φ. The curves deviate from the response for purely elastic expansion (φ = 0) at the critical pressure *p_c_*, as marked by purple squares. The decrease in pressure along the equilibrium branch is explained by the fact that although the volume is increasing, the normalized volume (*V*/*l*^3^) decreases, and thus motion along the pressure–volume curve (Fig. 1) changes direction. It is important to note that before onset of delamination the response is independent of whether the loading is applied via pressure control or volume control and adheres to the pressure–volume curve. At onset of delamination, a pressure controlled system would exhibit indefinite unstable expansion for any *p* > *p_c_*, whereas a volume controlled system would proceed along the descending branch.

The stable and unstable regimes of the system are shown on a phase diagram (Fig. 2). With the black curve indicating the critical pressure *p_c_*(φ)/μ; the blue and red shades distinguish stable and unstable regions, respectively. For strictly bonded interfaces (φ → 0), we recover the interfacial cavitation pressure; for vanishingly weak bonding, interfacial failure would dominate (φ → ∞). At the latter limit, the critical pressure scales as $1/\sqrt{\varphi }$. This result agrees with linear fracture models that are commonly used to model delamination phenomena (44–49). Markers on the phase diagram show experimental results from PIF experiments, as described in the next section. Overall, from this phase diagram, it is apparent that the interfacial cavitation limit serves as a tight upper-bound on interfacial failure for small φ. Additionally, by neglecting the highly nonlinear deformation that occurs at onset of failure, commonly employed models only apply for φ ≫ 1 and significantly overestimate the critical pressure (*p_c_*) for moderate to small values of φ. [Linear elasticity would predict *p_c_* → ∞ for φ → 0.]

## Interfacial cavitation explains PIF experiments

The competition between bulk and interfacial contributions in interfacial separation processes, and the nonlinear effects that ensue, have long been a hindrance on the development of methods for characterization of adhesion properties, where decoupling of the different effects is essential (59, 60). The PIF method was recently proposed to combat this issue (39). In the PIF experimental set-up, soft adhesives are compressed and then locally pressurised in a small region of an otherwise bonded interface (Fig. S1). As pressure increases the growth of an interfacial cavity is observed and appears as a circular delamination front, thus preserving axisymmetry, as assumed in our model. By identifying the pressure at initiation of instability (*p_c_*) and tracking the front propagation, this measurement technique pairs with a model that assumes a linear elastic response of the material to determine the interfacial toughness. PIF data is obtained for two materials systems: acrylic elastomers composed of poly(n-butyl)acrylate (PBA) networks cross-linked by ethylene glycol dimethacrylate (EGDMA), and commercial VHB tape, to explore a range of bulk and interfacial properties (Tables S1 and S2). The circular markers on the phase diagram represent experimental measurements of the critical pressure measured using this technique (Fig. 2). The corresponding material parameter (φ) is determined using a conventional probe-tack test (blue markers) or the PIF technique (green markers).

A remarkable agreement between the experimental measurements and the theoretical curve is observed. The theory serves as an upper-bound on the probe-tack data, whereas the PIF method appears to overestimate φ by underestimating the interface toughness. Quite interestingly, for higher interface toughness (lower φ), the experiments exhibit high values of critical pressure that exceed the bulk cavitation limit and approach the interfacial cavitation limit. This is a clear indication of highly nonlinear deformations that cannot be captured by linear models. Moreover, this demonstrates that interfacial cavitation, although a theoretical limit, is approached in synthetic material systems. Next, we will show that interfacial cavitation may be crucial also in determining the fate of natural systems, as we focus our attention to interfacial growth of bacterial biofilms.

## Cavitation to delamination transition observed in biofilm

Bacteria are often found on interfaces: at the site of a wound (61, 62); on a surgical implant (63); and in various chronic infections (64, 65). One cell embedded on an interface can multiply and form a biofilm consisting of tens of thousands of bacteria. This process inevitably deforms the surrounding medium, pushing it away from the substrate, and making room for reproduction. With most observations of biofilm growth conducted in absence of such resistance, on flat substrates (66–69), the confined growth of biofilm in 3D settings has only been observed in recent years (54) and has focused on the growth of biofilms as inclusions that are embedded in the bulk of a medium (53). However, less is known about the confined growth of biofilm at an interface; a situation which could be a better representation of in-vivo conditions (70). Our theory suggests that such growth could lead to mechanical instability; the particular mode of instability, and whether or not it is achieved, can influence the growth path of the biofilm and therefore alter its developmental trajectory.

Beyond its potential medical implications, observing the growth of biofilm at an interface also provides a unique opportunity to examine interfacial cavitation at the small scales. To this end, in our experimental system, isolated *Vibrio cholerae* bacteria are embedded at the interface between a glass substrate and soft agarose gels of varying stiffness. Starting from one cell, we use an adaptive imaging technique (71) that enables visualization of the global morphology over several orders of magnitude in volume (Figs. S2 and S3).

**Fig. 3.**
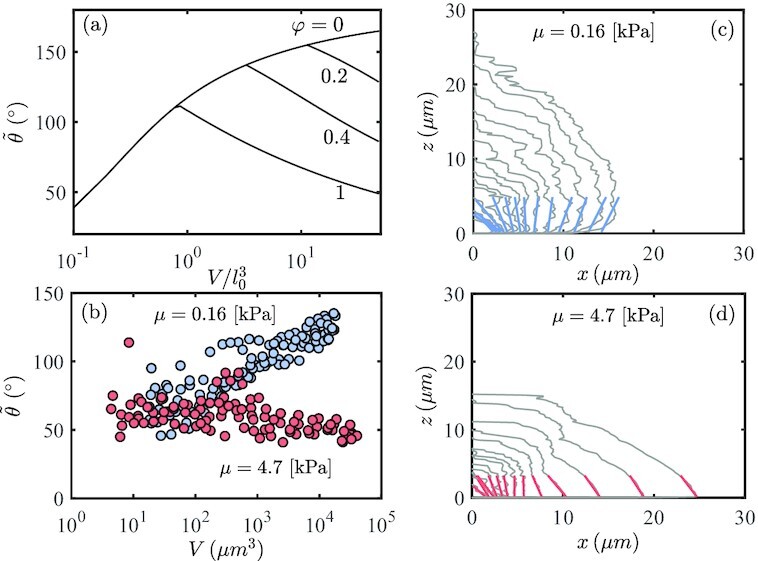
Evolution of interstitial biofilm exhibits a cavitation to delamination transition. (a) Theoretically predicted evolution of apparent angle, for increasing volume, shown for varying values of φ. (b) Experimentally measured apparent angle of biofilm of different volumes shown for two stiffnesses, μ = 0.16 and 4.7 [kPa], as marked by blue and red markers, respectively. (c, d) Shape evolution of a single biofilm, for each of the two stiffnesses. The colored lines represent the experimentally estimated apparent angles.

**Fig. 4.**
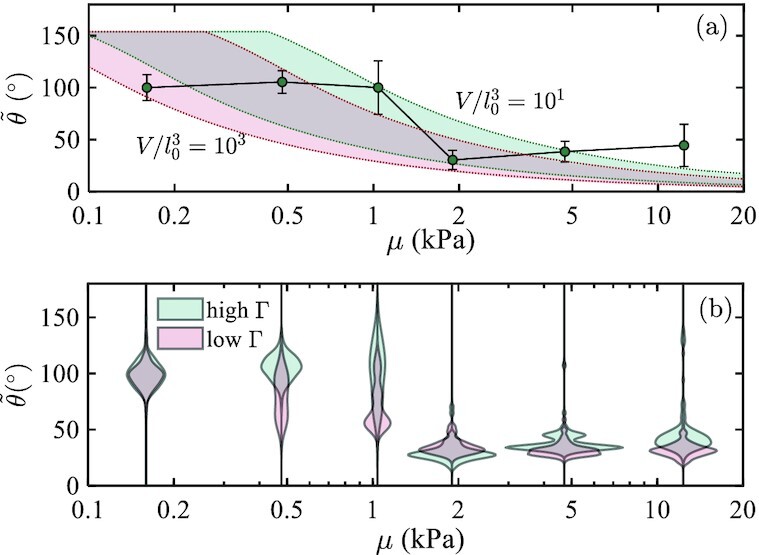
Influence of the interface toughness. (a) Theoretical prediction of apparent contact angle is shown for a range of mature volumes (as indicated by the shaded regions) for biofilm grown in confinements of different stiffness. Dashed lines correspond to two different volume expansions, *V* = (10^4^, 10^6^) μm^3^, with red and green corresponding to different interface toughness, Γ = (1.2, 2) × 10^−2^N/m, respectively, and using *l*_0_ = 10 μm as the initial defect size. For comparison, the green markers represent the experimentally measured value for the system with the higher Γ, and error bars show the SDs. (b) Experimentally measured mature apparent angles for two systems of different interface toughness are shown in the form of a violin plot, whereby the width of the cords are reflective of the probability density.

While biofilm have measurable solid-like mechanical properties (71), a recent study (53) has shown that on the timescale of growth, the persistent internal reorganization of cells in response to the mechanical confinement imparts the biofilm with fluid-like properties that dictate the evolution of its macroscopic shape. This implies that the reaction force between the biofilm and the confinement can be modeled as a hydrostatic pressure, as considered herein. Using this insight, we compare the theoretically predicted evolution of the interfacial cavity shapes with those observed in the biofilm system (Figs. 3 and 4). To quantify the shapes, we focus our attention to the *apparent contact angle* -- $\tilde{\theta }$, which we define as the internal angle at the intercept of the cavity contour with the interface. Since in the simulation the local angle can be highly influenced by discretization, for consistency we determine it as the uniquely defined intercept angle of a spheroid that intersects the interface at the same location and crosses through the peak location at *X* = 0. Accordingly, we show the numerically predicted evolution of the apparent contact angle, with increasing volume (log scale) in Fig. 3(a). If no delamination occurs the angle monotonically increases. At onset of delamination a clear transition to a decreasing trend is observed, and occurs earlier for increasing values of φ. Note that Fig. 3(a) is an alternative representation of the pressure–volume curves in Fig. 2, where the angle $\tilde{\theta }$ is used to describe the state of the cavity instead of *p*, which is not readily accessible in the biofilm system.

The theoretical trends are mirrored by our experimental observations of biofilm growth, as shown by comparing Fig. 3(a) with the experimental curves in Fig. 3(b). For lower stiffness of the confining gel (blue markers), the apparent angle continues to increase with increasing volume, thus approaching the interfacial cavitation limit. For the stiffer gel (red markers), the initial trend at small volumes resembles that of the softer gel, until a clear departure is observed (at *V* ∼ 300 μm^3^ with $\tilde{\theta }\sim 75^{\circ }$). The following monotonic decay indicates progression of delamination. A typical shape evolution of one biofilm for each of the two stiffnesses is shown in Fig. 3(c, d). The colored lines represent the apparent contact angle, which for the experimental curves, is estimated via a local linear fit.

Comparing the location of the transition point in Fig. 3(b) with the theorectical curves in Fig. 3(a) allows us to obtain an approximate measure of the initial defect size induced by the seed bacterium. Onset of delamination at $\tilde{\theta }\sim 75^{\circ }$ corresponds to the curve with φ ∼ 2.5, for which the transition occurs at the dimensionless volume $V/l_0^3 \approx 0.3$. Since the dimensional volume at the transition is *V* ∼ 300 μm^3^, we have that *l*_0_ ∼ 10 μm, corresponding to a small biofilm with tens of cells. Next, since the agarose stiffness is known (μ = 4.7[*kPa*]), along with φ and *l*_0_, we can estimate the interfacial toughness as Γ ∼ 2 × 10^−2^N/m. Note that the effective initial defect size (*l*_0_) is reflective of the length at which the biofilm is established and can be treated as a continuum body. In our experiments, we track the shapes of the biofilm starting from a single bacterium, as seen in Fig. 3(c, d).

Finally, we examine the influence of the interfacial toughness on biofilm shapes in Fig. 4. The apparent contact angles of hundreds of mature biofilms, grown under agarose gels of varying stiffnesses, are recorded for the system in Fig. 3, and also for a system in which we apply a treatment to the glass surface, to reduce the interface toughness (see the Supplementary Material for details). We define a mature biofilm as one that has grown for 12 to 16 h. After this growth period, we find that biofilm volumes can vary in the range *V* ∈ (10^4^, 10^6^) μm^3^. Assuming that the initial defect size remains of the same order (i.e. *l*_0_ ∼ 10 μm), we show the corresponding theoretically predicted range of apparent contact angles for two values of interface toughness, Γ = (1.2, 2) × 10^−2^ N/m, in Fig. 4(a). The latter corresponds to the results in Fig. 3 and is shaded green. The former represents a slight reduction in toughness, and is shaded red. The experimental results are shown in the form of a *violin plot* in Fig. 4(b), where the probability density of different apparent angles is represented by the width of the respective cords. For visual comparison, the average apparent angles for the system with higher interface toughness are also shown in Fig. 4(a), where error bars represent SDs. [Note that due to existing limitations in measurement techniques, we were not able to obtain an independent experimental measure of the Γ values, with sufficient resolution.]

Both the theoretical and the experimental results reveal that in softer environments, mature biofilms maintain large contact angles. The initial plateau at small stiffnesses suggests that the gel-substrate interface remains mostly intact. A clear transition to smaller angles is observed for the stiffer confinements. We attribute the more pronounced transition in the experimental data to the unstable nature of the delamination process, which promotes diffusion of solvent into the cavity, thus relaxing the volume constraint. By comparing results for the different values of interface toughness, both the theory and the experiments exhibit an earlier and smoother transition to the flat, delaminated shapes for the system with lower interface toughness.

Overall, we find that biofilms may deform the confining medium significantly, thus approaching the interfacial cavitation limit, before delamination is triggered. Both the material stiffness and the interface toughness play important roles in determining the fate these microscopic growing entities.

## Conclusions

In solids, cavitation that occurs at interfaces has received far less attention than bulk cavitation, which has become a well-established criteria for onset of failure in various materials, ranging from ductile metals to biological tissue. Though observations of interfacial failure have been reported, and can become dominant in heterogeneous materials, it has been primarily studied as a process of adhesive failure, which is length scale dependent. Our theoretical model shows that, in analogy to bulk cavitation, interfacial cavity expansion also arrives at a scale free asymptotic limit—the interfacial cavitation pressure – *p_ic_*/μ = 7/2, beyond which the expansion becomes unstable. Observations in two different experimental systems confirm that this theoretical limit is indeed approached, thus supporting the need for theory that can predict the highly nonlinear material response. Beyond cavitation, our theory extends to understand the propagation of delamination, thus providing a phase diagram that is useful to distinguish between stable and unstable regimes, and compares well with the experimental measurements. Given that interfacial failure is a ubiquitous phenomenon relevant to both synthetic and natural systems, we anticipate that by identifying the stability limits on their load bearing capacity will enable informed interpretation of experimental observations and can pave the way for design of more resilient heterogeneous material systems. Finally, this work is not without limitations, there is a breadth of opportunities to extend this work to examine the response for different constitutive models that incorporate strain stiffening, inelastic deformation, or compressibility, as well as extending the framework to capture the role of nonrigid substrates or inertia.

## Supplementary Material

pgac217_Supplemental_Files

## Data Availability

All data are included in the manuscript and/or Supplementary Material.
