# Supplementary material for: Interfacial cavitation
Source: PNAS Nexus. 2022 Oct 3;1(4):pgac217. doi: 10.1093/pnasnexus/pgac217 (PMC9802248; doi:10.1093/pnasnexus/pgac217)
Supplement: pgac217_Supplemental_Files [file pgac217_supplemental_files.zip › PNASNEXUS-PNASNEXUS-2022-00766-s03.pdf]

Interfacial Cavitation Supplementary Information

T. Henzel[a,1], **J. Nijjer**[b,1], **S. Chockalingam**[c,1], **H. Wahdat**[d], **A.J. Crosby**[d], **J. Yan**[b,*], **and T. Cohen**[a,e,*]

[a]Department of Civil and Environmental Engineering, Massachusetts Institute of Technology, Cambridge, MA 02139; [b]Molecular, Cellular and Developmental Biology , Yale University, New Haven, CT 06520; [c]Department of Aeronautics and Astronautics, Massachusetts Institute of Technology, Cambridge, MA 02139; [d]Polymer Science and Engineering Department, University of Massachusetts Amherst, Amherst, MA 01003, USA. ; [e]Department of Mechanical Engineering, Massachusetts Institute of Technology, Cambridge, MA 02139

[*] To whom correspondence should be addressed: T. Cohen (talco@mit.edu); J. Yan (jing.yan@yale.edu)
[1] These authors contributed equally.

S.1. Pressurized Interfacial Failure (PIF) Experimental Method

The PIF method was proposed by Wahdat et al. in[1]. Here we briefly describe the experimental system and its translation to compare with the theoretical predictions on this work.

A rigid annular probe with outer diameter R and inner diameter r initially pushes against an adhesive layer of thickness H, leaving a circular unadhered region at the interface that is enclosed by a much larger adhered region, as illustrated in Fig. S1. Ideally, for the purpose of the present work, the dimensions are such that $r/R, r/H \ll 1$, and the applied compressive force, f, translates to an approximately uniform hydrostatic stress field $\sigma_h = f/\pi R^2$ in the region beneath the probe, if the initial internal pressure of the probe, p, is set equal to the applied hydrostatic stress (i.e. $p = \sigma$). In our set-up we have $R = 1000\mu$m and $r = 100\mu$m. Our adhesive layers are fabricated with $h = 1000\mu$m or 300μm. The initial compression is chosen to ensure that full adhesive contact is achieved. The displacement of the probe is then fixed and the internal pressure (p) is gradually increased by compressing the gas in the volume enclosed by the inner walls of the probe and the sample. The critical pressure, $\hat{p}_c$, is identified once delamination initiates. In our experimental systems this can be directly inferred from the change in force f that is measured simultaneously. As explained in the main text and in[2], the initially applied hydrostatic stress has an additive effect on the applied critical pressure, and thus to compare with predictions in Fig. 2 of the main text we write

$$p_c = \hat{p}_c - \sigma \qquad [1]$$

and the initial defect length is $l_0 = 2r$.

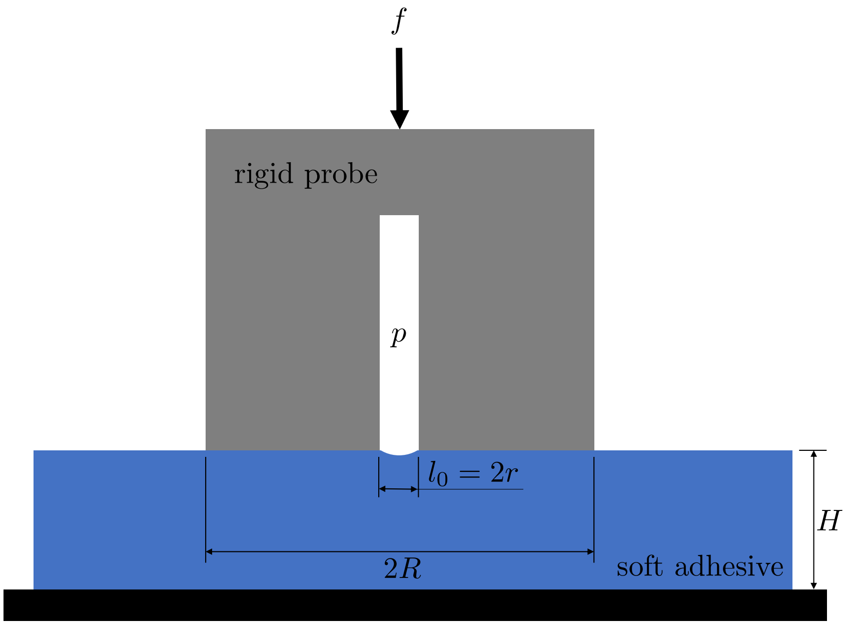

Fig. S1. Schematic illustration of the PIF set-up.

S.2. Bulk and Interfacial Properties of Adhesives Used in PIF Experiments

In the PIF experiments we use two material sytems:

(1) Poly(n-butyl)acrylate (PBA) networks cross-linked by ethylene glycol dimethacrylate (EGDMA). We denote by ϕ the weight fraction of the total monomer mass of the EGDMA cross-linker. All PBA samples were prepared from UV-curable formulations. The preparation protocol is described in[1].

(2) Commercially available VHB Tape (3M, VHB4910, thickness = 1 mm).

The mechanical properties of these materials have been reported in[1] and included here for completeness (Table S.1). The shear modulus, μ, was inferred from force displacement curves in the initial compression of the sample with the PIF probe. The interfacial toughnesses were inferred from the propagation of the delamination using the linear model described in[1] (Γ_{PIF}), and from the conventional sphere-probe tack test (Γ_{SPT}). All materials are assumed to be incompressible.

Material	ϕ	μ(kPa)	Γ_{PIF}(J/m^2)	Γ_{SPT}(J/m^2)
PBA	0.25	14.33(1.33)	1.508	0.563
PBA	0.25	14.33(1.33)	1.687	0.563
PBA	0.25	11.67(1.33)	2.103	0.601
PBA	0.25	11.67(1.33)	1.953	0.601
PBA	0.5	36(2.33)	0.728	0.776
PBA	0.5	36(2.33)	1.001	0.776
PBA	0.5	31.33(0.33)	1.235	0.656
PBA	0.5	31.33(0.33)	1.824	0.656
PBA	1	100(6.67)	1.743	0.052
PBA	1	300(6.67)	0.712	0.052
PBA	1	89.33(3)	0.503	0.050
PBA	1	89.33(3)	0.342	0.050
VHB	-	27(1)	5.924	1.670
VHB	-	27(1)	5.478	1.670

Table S.1. Mechanical properties of adhesive layers. Values in parentheses represent the standard deviation. Additional details on the experimental methods are found in[1] and in the references therein.

S.3. Critical Pressure Measurements Obtained by PIF Method

In the present paper, to compare the PIF results with the theoretical predictions for expansion of interfacial cavities, we focus our attention to the critical pressure Eq. (1). The experimental data used to create Fig. 2, in the main text, is provided in Table S.2. For the comparison with the theoretical results in Fig. 2, φ is calculated for each sample using the interfacial toughness and shear modulus values from Table S.1, and l_0=0.2 mm.

S.4. Experimental Details of the Biofilm Growth Experiments

Sample preparation: The bacterial strain used for biofilm experiments was a derivative of the *Vibrio cholerae* strain C6706 with a point mutation in the *vpvC* gene, which resulted in upregulated biofilm production. We further deleted the genes *rbmA*, *bap1*, and *rbmC*, to exclude the effects of cell-substrate and cell-cell adhesion. To perform the experiments, the bacteria were first grown overnight in Lysogeny broth at 37°C under shaken conditions. The overnight culture was then diluted 30x into M9 media supplemented with 0.5% glucose, 2 mM $MgSO_4$ and 100 μM

Please provide details of author contributions here.

Please declare any conflict of interest here.

[1] T. Henzel and S. Chockalingam contributed equally to this work.

Material	ϕ	$H(\mu$m)	σ(kPa)	$\hat{p}_c$(kPa)	p_c(kPa)
PBA	0.25	300	4.983	26.090	21.107
PBA	0.25	300	21.068	41.327	20.259
PBA	0.25	1000	11.086	32.788	21.702
PBA	0.25	1000	1.694	25.767	24.073
PBA	0.5	300	13.867	38.705	24.838
PBA	0.5	300	21.392	46.387	24.995
PBA	0.5	1000	14.640	41.251	26.611
PBA	0.5	1000	22.973	56.556	33.584
PBA	1	300	95.040	137.620	42.580
PBA	1	300	30.443	72.941	42.498
PBA	1	1000	16.215	45.522	29.307
PBA	1	1000	48.002	66.132	18.130
VHB	-	1000	9.152	91.603	82.452
VHB	-	1000	54.289	114.927	60.638

Table S.2. Measured applied hydrostatic stress, and critical pressures, for samples of different material compositions and with different initial thickness.

$CaCl_2$ and grown at 30°C for 1-2 hours under shaken conditions. This culture was then diluted to an OD_{600} of $1-3\times10^{-3}$, and a 1 μL droplet of the diluted solution was placed in the center of a glass-bottomed 96 well plate (MatTek). The droplet was then covered with 20 μL of molten agarose gel of concentration 0.2-1%, encasing the cells. Upon cooling, 200 μL of the above-mentioned supplemented M9 media was added. The plates were incubated at 30°C and either imaged periodically or after 12-16 hrs.

Characterization of agarose stiffness: The stiffness of the agarose gels was measured using a shear rheometer (Anton Paar Physica).

Surface treatment: To reduce the interfacial toughness between the agarose gel and the glass substrate, we treated the substrate using vapor deposition of silane by enclosing the 96 well plate in a box with a 1 mL reservoir of 3-aminopropyltriethoxysilane (Sigma) for 16 hrs.

Imaging: Images were taken using a confocal spinning disk unit (Yokogawa CSU-W1; Nikon Eclipse Ti2; Photometrics Prime BSI). For time-lapse imaging, a 100x silicon oil immersion objective was used to image biofilms at z-intervals of 0.13 or 0.195 μm. For endpoint imaging of mature biofilm contact angles, a 60x water immersion objective was used to image the bottom 5 μm of the biofilms at z-intervals of 0.4 μm. In the latter case, 100 fields of view were imaged resulting in an average of 89 (range 27-140) unique biofilm measurements for each concentration.

Image analysis: Biofilm images were de-noised and deconvolved using Huygens SVI software and binarized using Otsu thresholding in MATLAB (2018a). For each slice in the confocal image, a convex hull, which encompassed all binarized pixels in each biofilm, was found. The area of the convex hull was used to estimate the height-dependent cross-sectional area of the biofilm $A(z)$ from which the effective radius was calculated as $r(z)=(A/\pi)^{1/2}$. The contact angle θ was then found by fitting a linear slope in the bottom 5 μm of the biofilm,

$$\theta = \frac{180}{\pi}\left(\tan^{-1}\left(\frac{\mathrm{d}r}{\mathrm{d}z}\right)+\frac{\pi}{2}\right)\ \text{[deg]}.$$

Biofilm growth: Images of biofilms at different times throughout the growth process, as referred to in Fig. 3. of the main text, can be found in Figs. S2 and S3.

1. Wahdat H, Zhang C, Chan N, Crosby AJ (2022) Pressurized interfacial failure of soft adhesives. *Soft Matter.*
2. Henzel T, Senthilnathan C, Cohen T (2022) A reciprocal theorem for finite deformations in incompressible bodies. *arXiv preprint arXiv:2201.08338.*

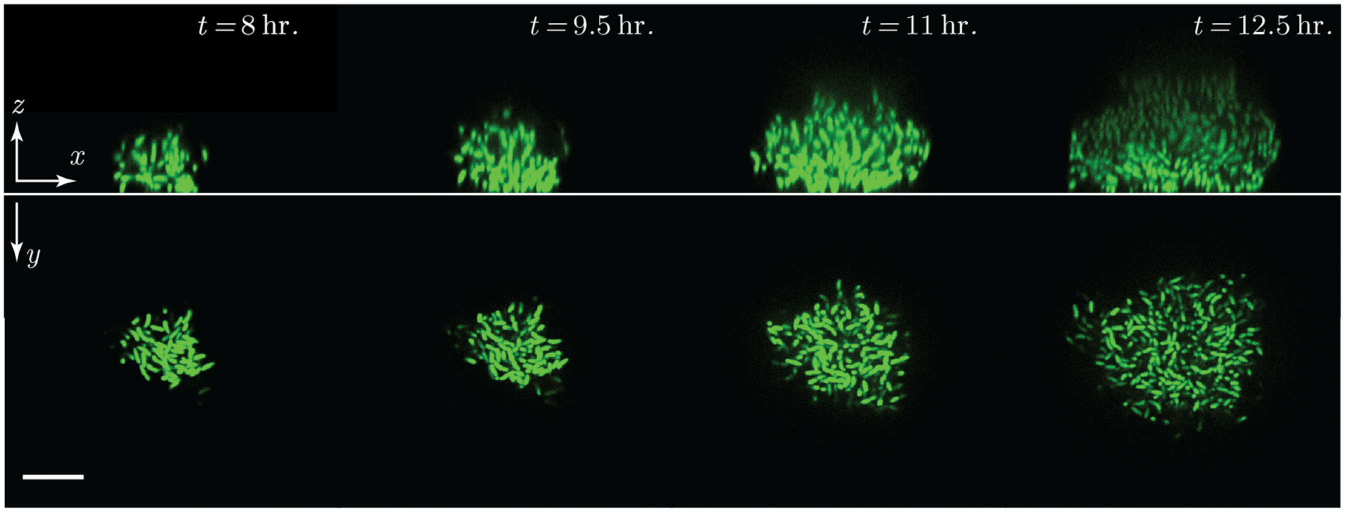

Fig. S2. Confocal images showing the growth of a single biofilm at different times throughout the growth process, at single cell resolution. The biofilm is grown under the confinement of an agrose gel of stiffness μ=0.16 (kPa). Scale bar is 10μm.

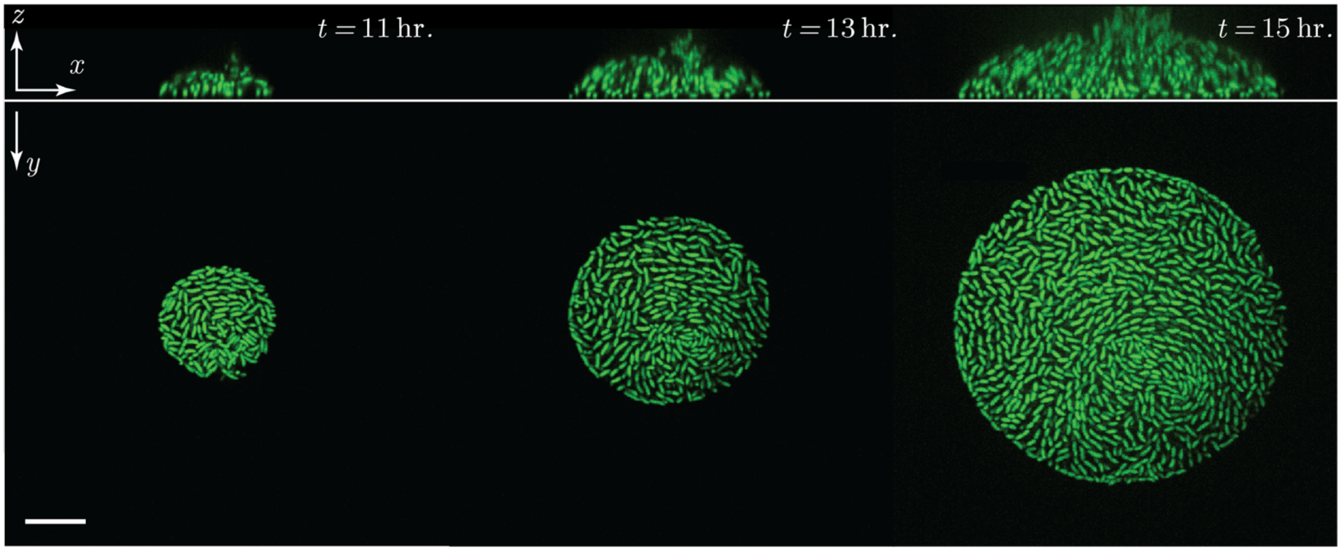

Fig. S3. Confocal images showing the growth of a single biofilm at different times throughout the growth process, at single cell resolution. The biofilm is grown under the confinement of an agrose gel of stiffness μ=4.7 (kPa). Scale bar is 10μm.
